# Association of Serum Cystatin C with Stroke Morbidity and All-Cause and Cardio-Cerebrovascular Mortality: Evidence from the NHANES

**DOI:** 10.3390/healthcare13172137

**Published:** 2025-08-27

**Authors:** Si Hu, Guoqiang Zhang, Wei Zhou, Yi Hu, Jingwei Zheng, Fei Liu, Zhijie Jiang, Xudan Shi, Kaiyang Shao, Liang Xu

**Affiliations:** 1 Department of Neurosurgery, Second Affiliated Hospital, Zhejiang University School of Medicine, Hangzhou 310009, China; hs930207@163.com (S.H.); drzgq@zju.edu.cn (G.Z.); drstrange1994@zju.edu.cn (J.Z.); 22318505@zju.edu.cn (Z.J.); 2 Department of Neurosurgery, Affiliated Huzhou FuYin Hospital of Huzhou University, Huzhou 313000, China; hy181224@163.com; 3 Clinical Research Center for Neurological Diseases of Zhejiang Province, Hangzhou 310009, China; 2320053@zju.edu.cn (F.L.); shixudan@hotmail.com (X.S.); shaokaiyang@zju.edu.cn (K.S.); 4 Department of Cardiology, Affiliated Huzhou FuYin Hospital of Huzhou University, Huzhou 313000, China; chizha22222@163.com; 5 Neuroscience Intensive Care Unit, Second Affiliated Hospital, Zhejiang University School of Medicine, Hangzhou 310009, China; 6 Department of Anesthesiology, Second Affiliated Hospital, Zhejiang University School of Medicine, Hangzhou 310009, China; 7 Department of Health Management Center, Second Affiliated Hospital, Zhejiang University School of Medicine, Hangzhou 310009, China

**Keywords:** cystatin C, NHANES, cardiovascular, cerebrovascular, morbidity, mortality

## Abstract

**Background:** Serum cystatin C is a promising biomarker for vascular risk, yet its nonlinear dose–response relationships and prognostic value in general populations remain unclear, particularly for stroke-specific outcomes. **Methods:** This study utilized data from the National Health and Nutrition Examination Survey (NHANES) conducted in 1999–2002 cycles. A total of 11,610 participants were included in the primary analysis examining the cross-sectional association between cystatin C and stroke morbidity, using multivariate logistic regression models and odds ratios (ORs). Analyses utilized complete-case data (n = 11,610 for morbidity; n = 11,598 for mortality). Subsequently, 11,598 adults were retained for mortality endpoint analyses, which focused on the longitudinal association between cystatin C and stroke mortality, using cause-specific weighted multivariable Cox models and ratios (HRs). Restricted cubic splines identified nonlinear thresholds, and piecewise regression quantified risk gradients. Models were adjusted for sociodemographic/clinical/behavioral confounders. **Results:** Serum cystatin C exhibited a nonlinear dose–response relationship with stroke morbidity (*p* for nonlinear < 0.001), with an inflection point at 1.24 mg/L; below this threshold, each 0.1 mg/L increase conferred 13.84-fold higher odds (95% CI: 7.11–27.03, *p* < 0.001). For mortality, nonlinear thresholds were identified at 1.24 mg/L for all-cause/cause-specific mortality (HR = 6.73–10.60 per 0.1 mg/L increase, *p* < 0.001) and 1.81 mg/L for stroke-specific mortality. Conversely, cerebrovascular mortality demonstrated a linear association (HR = 1.43 per 1 mg/L increase, *p* = 0.008), though cystatin C independently predicted risk (HR = 1.38/continuous, *p* = 0.034 in fully adjusted models). **Conclusions:** This study identifies serum cystatin C as an independent predictor after full adjustment of stroke morbidity and all-cause and cardio-cerebrovascular mortality. Consequently, cystatin C emerges as a dual-purpose biomarker for early vascular injury detection in subclinical populations and integrated mortality risk stratification. Future research should validate these thresholds in prospective neuroimaging-confirmed cohorts and investigate interventions targeting cystatin C pathways to optimize preventive strategies.

## 1. Introduction

Globally, stroke remains the second leading cause of death and the third leading cause of disability, accounting for 11.6% of total deaths and 5.7% of disability-adjusted life years (DALYs) worldwide, as estimated by the 2019 Global Burden of Disease (GBD) report [1]. Epidemiological projections reveal an alarming trajectory: by 2050, global stroke survivors may exceed 200 million, with incident cases doubling to 25 million annually and DALYs approaching 300 million [1]. Disparities in stroke burden are pronounced in high-incidence regions. For instance, China’s age-standardized incidence rate (ASIR) exceeds the global average by 34%, while out-of-pocket medical expenditures for stroke hospitalization in northeastern provinces (42.5% of total costs) surpass the WHO (World Health Organization)-defined catastrophic health expenditure threshold by twofold [2,3,4]. Concurrently, the United States experiences a stroke event every 40 s, with direct medical costs projected to exceed $100 billion by 2035 and in-hospital mortality rates persisting at 19.6–21.3% [5,6]. This escalating disease burden necessitates precise identification of modifiable risk factors and their pathophysiological synergies to inform primary prevention strategies. Serum biomarkers, characterized by standardized quantification and clinical accessibility, hold potential for enhancing risk prediction models and guiding personalized interventions.

Cystatin C, a cysteine protease inhibitor constitutively expressed by nucleated cells, is a superior glomerular filtration rate (GFR) estimator due to its minimal confounding by demographic variables, particularly in early-stage renal dysfunction detection [7,8]. Emerging evidence suggests that cystatin C may be involved in extrarenal pathologies, including subclinical atherosclerosis, endothelial dysfunction, and neuroinflammatory cascades [9,10]. Regarding cerebrovascular mortality, meta-analytic evidence indicates significantly elevated serum cystatin C levels in non-survivors of acute ischemic stroke compared to survivors [11]. Cohort studies further corroborate these findings, reporting associations between elevated cystatin C and increased post-stroke mortality [12,13]. Though inconsistencies persist due to heterogeneous adjustment for renal function and comorbidities. The Tromsø Study, for instance, failed to demonstrate an independent association between cystatin C and incident ischemic stroke after multivariable adjustment, although it did identify cystatin C as a mortality risk factor specifically in women (adjusted HR: 1.38 for the highest quartile) [14]. Kim et al. identified cystatin C as a predictor of early neurological deterioration in ischemic stroke [15], while its independent prognostic value for cerebrovascular-specific mortality remains inadequately characterized in population-based cohorts. While observational studies have associated elevated cystatin C with an increased risk of stroke [16,17], the prognostic value of cystatin C for cardio-cerebrovascular mortality remains inconclusive due to methodological heterogeneity. Three critical limitations constrain current interpretations: (1) the predominant focus on high-risk subgroups (e.g., chronic kidney disease cohorts) limits external validity; (2) insufficient characterization of dose–response gradients and joint associations with mortality endpoints; and (3) residual confounding from socioeconomic determinants (e.g., health literacy) and comorbid conditions in multivariate analyses.

To address these gaps, we analyzed data from the National Health and Nutrition Examination Survey (NHANES), a population-based cohort with ethnically diverse representation, to evaluate serum cystatin C’s independent associations with stroke morbidity, all-cause mortality, and cardio-cerebrovascular mortality. Utilizing survey-weighted multivariable regression models and restricted cubic splines to identify nonlinear relationships, we quantified exposure–response relationships across cystatin C concentrations, adjusted for sociodemographic, clinical, and behavioral confounders. We aimed to provide epidemiological evidence for optimizing risk stratification thresholds and corroborate cystatin C’s prognostic value in general populations—a significant advancement given the current reliance on selective clinical samples.

## 2. Materials and Methods

### 2.1. Study Design and Participants

Data from the 1999–2002 cycles of NHANES, a nationally representative surveillance program administered by the Centers for Disease Control and Prevention (CDC), were used to investigate the cross-sectional relationship with stroke morbidity and the longitudinal relationship with stroke mortality. NHANES employs multistage stratified probability sampling to recruit non-institutionalized U.S. civilians, collecting demographic, clinical, and biochemical data through structured interviews, standardized physical examinations, and laboratory assays. The study protocol was approved by the National Center for Health Statistics (NCHS) Ethics Review Board, and written informed consent was obtained from all participants. Publicly available data were obtained from the NHANES repository (https://www.cdc.gov/nchs/nhanes/ (accessed on 1 August 2024)).

**Figure 1:**
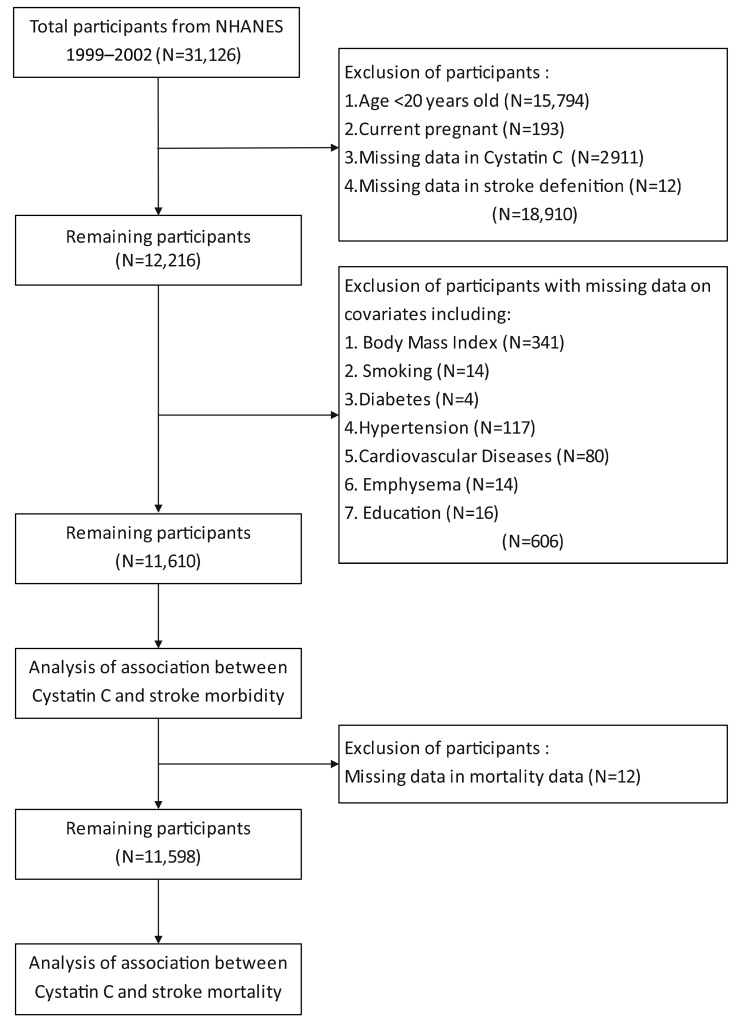
Flowchart of participant selection.

From a cohort of 31,126 eligible participants (1999–2002), we systematically excluded 18,910 individuals due to age < 20 years (n = 15,794), current pregnancy (n = 193), missing data in cystatin C (n = 2911), or missing data in stroke definition (n = 12). Subsequently, 606 participants were excluded for incomplete covariate data, including body mass index (BMI) (n = 341), smoking status (n = 14), diabetes (n = 4), hypertension (n = 117), cardiovascular diseases (n = 80), emphysema (n = 14), and education level (n = 16). Participants with incomplete covariate data were excluded to maintain a complete-case framework. The remaining 11,610 participants were included in the primary analysis of cystatin C-stroke morbidity associations. After further excluding 12 individuals with unavailable mortality follow-up, the final cohort of 11,598 adults was retained for mortality endpoint analyses. The complete exclusion process is depicted in Figure 1.

### 2.2. Cystatin C and Stroke Ascertainment

Cystatin C was measured in serum using a particle-enhanced immunonephelometric assay (Siemens Healthcare Diagnostics) on a Dimension Vista 1500 analyzer (Siemens, Forchheim, Germany), consistent with NHANES protocols and prior standardized methods [7,8,18]. The assay utilized N Latex Cystatin C reagents (Dade Behring) calibrated per manufacturer specifications. Detailed measurements can be found at https://www.cdc.gov/Nchs/Nhanes/1999–2000/LAB18.htm (accessed on 1 August 2024).

Stroke cases were identified through validated self-reported physician diagnoses. Participants responded to the standardized item: “Has a doctor or other healthcare professional ever diagnosed you with a stroke?” (lifetime history) administered via Computer-Assisted Personal Interviewing (CAPI). The CAPI system integrated automated range checks, real-time error alerts, and medical term definitions to minimize interviewer bias. Anomalous responses triggered immediate verification protocols before data finalization.

### 2.3. Mortality Outcomes

All-cause and cause-specific mortality (cardiovascular and cerebrovascular) were ascertained through linkage with the National Death Index (NDI) using deterministic matching algorithms based on Social Security numbers, birthdates, and demographic identifiers. NDI linkage used Social Security Number, birth date, sex, and race/ethnicity in stepwise exact matching protocols per NCHS specifications (https://www.cdc.gov/nchs/data/ndi/2024-NDI-User-Guide.pdf (accessed on 1 August 2024)). NDI linkage had complete cause-of-death data for 98.7% of deceased participants; 15 records with undetermined causes were excluded per NCHS protocols. The follow-up extended from the baseline interview until death or 31 December 2019, whichever occurred first. Cardiovascular deaths were classified using ICD-10 codes I00–I78, and cerebrovascular deaths as I60–I69, consistent with NCHS reporting standards. A competing event in this study was defined as cardiovascular, cerebrovascular, or non-cardio-cerebrovascular mortality, whichever occurred first in the follow-up period.

### 2.4. Covariates

The analysis incorporated covariates that addressed sociodemographic, clinical, and behavioral confounders. Sociodemographic variables included age (continuous), gender (male/female), race/ethnicity (categorized as Mexican American, non-Hispanic Black, non-Hispanic White, other Hispanic, or other/multiracial), and the family income-to-poverty ratio. Educational attainment was classified as less than high school, high school, or more than high school. Clinical variables comprised BMI (kg/m^2^), smoking status, and diabetes status. Hypertension, coronary heart disease (CHD), congestive heart failure (CHF), emphysema, and chronic kidney disease (CKD) were also included.

Anthropometric measurements (weight and height) were obtained from standardized physical examinations conducted in mobile assessment centers. Participants with incomplete covariate data were excluded to maintain a complete-case analytical framework.

### 2.5. Statistical Analysis

Continuous variables were expressed as weighted means ± standard deviations (SD) and categorical variables as proportions (%), accounting for the complex sampling design of NHANES. Weighted logistic regression and chi-square tests were employed to determine *p*-values for continuous and categorical variables, respectively. Serum cystatin C was analyzed as both continuous (per 1 mg/L increment) and categorical (quartiles) variables. The continuous form assesses linear dose–response relationships, while quartiles evaluate nonlinear effects. Serum cystatin C was categorized into quartiles (Q1: ≤25th percentile; Q2: 25–50th; Q3: 50–75th; Q4: >75th percentile) to evaluate nonlinear effects. Multivariable logistic regression models were constructed to assess the associations between serum cystatin C quartiles (Q1–Q4, with Q1 as the reference) and stroke morbidity through three sequential adjustments: unadjusted (Model 1), minimally adjusted for age, sex, and race/ethnicity (Model 2), and fully adjusted for sociodemographic, clinical, and behavioral covariates (Model 3). The goodness of fit was evaluated using the Akaike Information Criterion (AIC) and Bayesian Information Criterion (BIC), with lower values suggesting better model fit. Additionally, optimal clinical cut-off points were determined using Youden’s index (J = sensitivity + specificity − 1) via receiver operating characteristic analysis.

Nonlinear associations were investigated using restricted cubic splines (RCS). We used a recursive algorithm implemented in the ggrcs R package to calculate inflection points. A two-segment cause-specific Cox proportional hazards model was then used on either side of each inflection point to investigate the association between cystatin C and the risk of stroke morbidity and mortality outcomes.

For mortality outcomes, cause-specific hazard ratios (HRs) were estimated using three adjustment levels. Kaplan–Meier curves were employed to compare survival probabilities across cystatin C quartiles for all-cause mortality and all-cause mortality in patients with stroke. To avoid upward-biased survival estimates, we also generated the nonparametric inverse probability–weighted cumulative incidence function curves by considering cardiovascular mortality, cerebrovascular mortality, and non-cardio-cerebrovascular mortality as competing risks [19]. We further implemented Gray’s test for the equivalence of the cumulative incidence function to compare weighted cumulative incidence by cystatin C quartiles using the R package tidycmprsk (version 1.1.0).

To address sparse event counts in cardiovascular mortality (n = 850) and cerebrovascular mortality (n = 169), Firth’s penalized-likelihood Cox regression was employed to mitigate small-sample bias. Additionally, clinical representativeness of sparse subgroups was assessed through direct comparison with larger mortality groups. Sensitivity analyses confirmed consistency between approaches. Subgroup analyses were conducted to investigate heterogeneity across strata of gender, race/ethnicity, education, BMI, hypertension, diabetes, CKD, and smoking status. All analyses incorporated survey weights and adjusted for clustering and stratification effects. All *p*-values were calculated using survey-weighted methods (svydesign function from the survey package in R) to account for NHANES’ complex sampling design, including clustering and stratification effects. Statistical significance was defined as a two-tailed *p* < 0.05. Data processing and modeling were implemented using R version 4.3.1.

## 3. Results

### 3.1. Baseline Characteristics

The analytical cohort consisted of 11,610 adults (362 stroke cases; 11,248 non-cases), with a weighted mean age of 49.73 ± 18.76 years. Females comprised 51.69% of the population. The racial/ethnic distribution was as follows: non-Hispanic White (51.90%), non-Hispanic Black (17.90%), Mexican American (22.19%), other Hispanic (4.52%), and multiracial/other (3.47%). The mean serum cystatin C levels were 0.82 ± 0.36 mg/L, and the average BMI was 28.36 ± 6.19 kg/m^2^. Baseline characteristics are presented in Table 1.

Distinctive characteristics emerged between the stroke and non-stroke groups. Stroke patients were significantly older (67.57 ± 14.37 years vs. 49.15 ± 18.60 years; *p* < 0.001), with a higher proportion of non-Hispanic White individuals (56.63% vs. 51.75%) and non-Hispanic Black individuals (19.89% vs. 17.85%; *p* = 0.031). Socioeconomic disparities were evident through both educational attainment (less than high school education: 45.58% vs. 30.76%; *p* < 0.001) and income measures (PIR: 2.20 ± 1.44 vs. 2.67 ± 1.61; *p* < 0.001). Additionally, smoking prevalence was significantly higher in stroke cases (55.5% vs. 48.8%; *p* = 0.012).

Clinically, stroke patients exhibited significantly elevated cystatin C levels (1.09 ± 0.55 mg/L vs. 0.81 ± 0.35 mg/L; *p* < 0.001) and a higher burden of comorbidities, including BMI (29.32 ± 6.07 vs. 28.33 ± 6.19 kg/m^2^), hypertension (71.55% vs. 30.38%), diabetes (23.20% vs. 9.10%), CHD (18.51% vs. 3.95%), CHF (15.75% vs. 2.52%), emphysema (6.63% vs. 1.73%), and CKD (9.67% vs. 2.41%) (all *p* < 0.001). No sex-based differences were observed (female: 48.07% vs. 51.80%; *p* = 0.165). Cystatin C quartile distributions diverged substantially, with 62.43% of stroke cases concentrated in Q4 compared to 23.72% of non-cases (*p* < 0.001).

### 3.2. Association Between Cystatin C and Stroke Morbidity

Serum cystatin C concentrations exhibited a graded positive association with stroke morbidity across multivariable-adjusted models (Table 2). Each 1 mg/L increment in cystatin C was associated with an unadjusted odds ratio (OR) of 2.28 (95% CI: 1.66–3.12; *p* < 0.05). Adjustment for demographic factors attenuated the association (OR = 1.65, 95% CI: 1.39–1.95; *p* < 0.05), with further attenuation following adjustment for education level, family income-to-poverty ratio, BMI, hypertension, diabetes, CKD, and smoking (OR = 1.27, 95% CI: 1.02–1.58; *p* = 0.024). Quartile-based analyses revealed a dose-dependent relationship, with the highest quartile (Q4) exhibiting 4.59-fold elevated odds compared to the lowest quartile (Q1) in the fully adjusted model (95% CI: 2.10–10.06; *p* < 0.001). AIC/BIC comparisons (Supplementary Table S8) confirmed that categorical quartiles (Model 3 AIC = 1968.68) outperformed continuous specifications (AIC = 1991.23), supporting nonlinear risk stratification. Using the clinical reference range (0.51–0.98 mg/L) established by Finney et al. [18], we further compared stroke risk between participants with elevated cystatin C (>0.98 mg/L, n = 1743) and those with normal levels (≤0.98 mg/L, n = 9855). In fully adjusted models, elevated cystatin C was associated with a 2.03-fold higher odds of stroke (OR = 2.03, 95% CI: 1.46–2.82; *p* < 0.001; Supplementary Table S1). Youden’s index analysis identified 0.885 mg/L as the optimal clinical cut-off, with values > 0.885 mg/L conferring 2.20-fold higher stroke risk (OR = 2.20, 95% CI: 1.51–3.21; *p* < 0.001; Supplementary Table S2). Supplementary Figure S1A displays the ROC curve for cystatin C’s prediction of stroke morbidity. At the optimal cutoff of 0.885 mg/L, sensitivity was 72.1% and specificity was 67.7% with AUC = 0.758.

**Figure 2:**
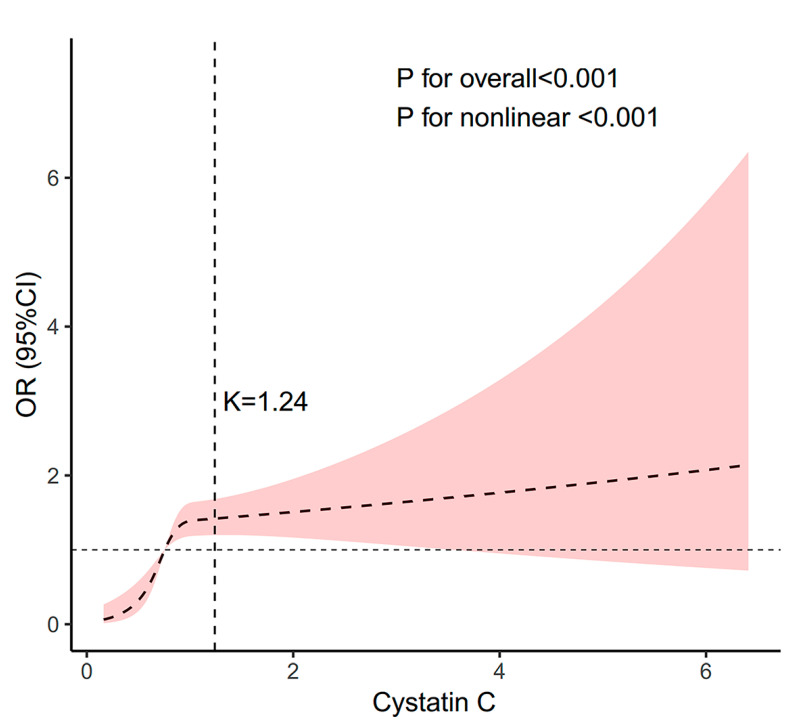
Association between cystatin C and stroke morbidity. Nonlinear association between serum cystatin C and stroke morbidity below 1.24 mg/L (*p* for nonlinear < 0.001) with no significant linear association above this threshold (*p* = 0.872, Table 3). Adjusted for age, gender, and race. The solid line and purple area represent the estimated values and their corresponding 95% CIs, respectively.

RCS analysis revealed a nonlinear dose-response pattern (*p* for nonlinear < 0.001), with an inflection point at 1.24 mg/L (Figure 2, Table 3). Below this threshold, each 0.1 mg/L increase in cystatin C was associated with a 13.84-fold elevation in stroke risk (OR = 13.84, 95% CI: 7.11–27.03; *p* < 0.001), whereas no significant association was observed above 1.24 mg/L (OR = 1.02, 95% CI: 0.78–1.26; *p* = 0.872). The 1.24 mg/L threshold demarcates critical risk phases: subthreshold concentrations (<1.24 mg/L) exhibit exponential stroke risk elevation, while suprathreshold levels show no significant trend.

**Figure 3:**
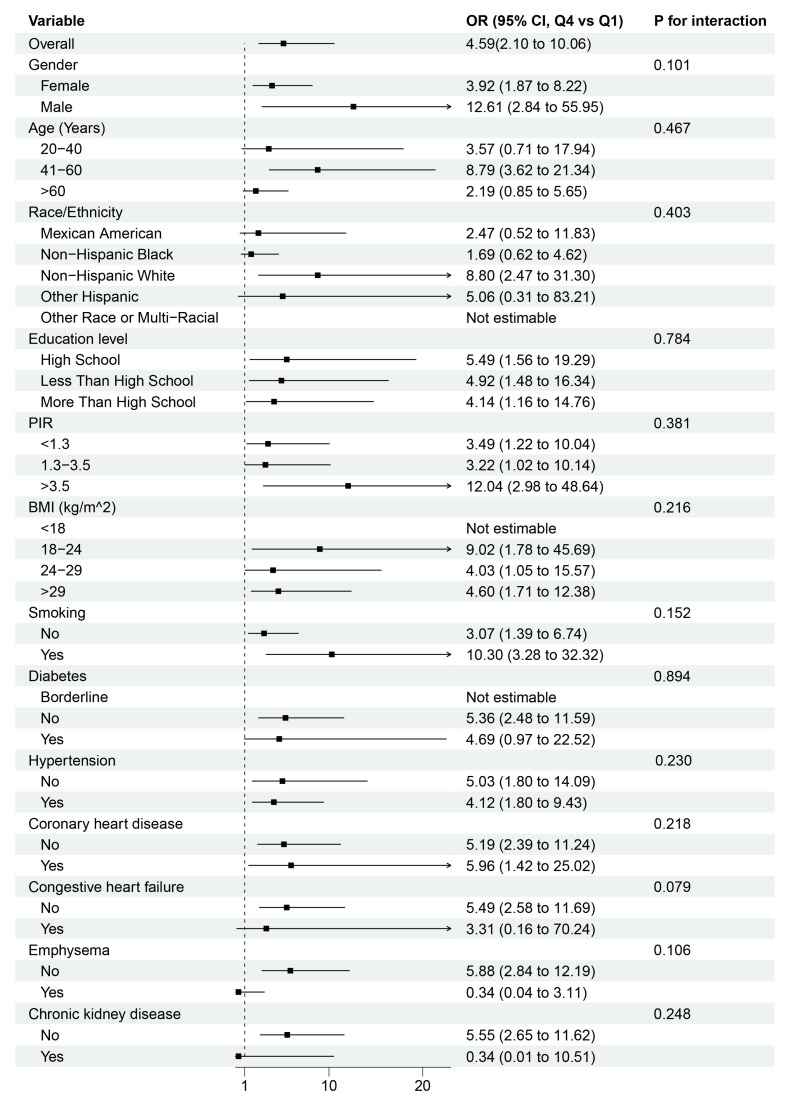
Subgroup analysis of the association between cystatin C quartiles (Q4 vs. Q1) and stroke morbidity. Adjusted for age, gender, race/ethnicity, educational level, family income-to-poverty ratio, BMI, smoking status, hypertension, diabetes, and chronic kidney disease. PIR, family income-to-poverty ratio, BMI, body mass index.

Subgroup analyses stratified by clinical and demographic characteristics revealed heterogeneous associations between cystatin C levels and stroke risk (Figure 3). Subgroup analyses of the association between cystatin C quartiles (Q4 vs. Q1) and stroke risk are presented in Figure 3. The overall association was significant (OR = 4.59, 95% CI: 2.10–10.06). Formal interaction tests for comorbidities showed no statistically significant effect modification (all *p* for interaction > 0.05), though point estimates revealed clinically meaningful gradients. Participants without CKD exhibited substantially higher risk (OR = 5.55, 95% CI: 2.65–11.62) compared to those with CKD (OR = 0.34, 95% CI: 0.01–10.51). Similarly, non-CHF subgroups showed elevated odds (OR = 5.49, 95% CI: 2.58–11.69) versus CHF subgroups. For coronary heart disease, both non-CHD (OR = 5.19, 95% CI: 2.39–11.24) and CHD (OR = 5.96, 95% CI: 1.42–25.02) subgroups demonstrated comparable risk elevations.

### 3.3. Association Between Cystatin C and Mortality

Among 11,598 participants with complete mortality data, elevated serum cystatin C quartiles (Q2–Q4) were associated with progressively adverse demographic and clinical profiles compared to Q1 (Table 4). Mean cystatin C levels ranged from 0.59 ± 0.06 mg/L (Q1) to 1.16 ± 0.57 mg/L (Q4; *p* < 0.001). Participants in Q4 were older (66.09 ± 16.08 vs. 38.05 ± 13.00 years; *p* < 0.001), predominantly male (52.82% vs. 31.96%), and exhibited a higher prevalence of hypertension (53.93% vs. 16.43%), diabetes (16.68% vs. 6.19%), CHD (10.66% vs. 0.89%), and CKD (6.72% vs. 1.03%; all *p* < 0.001). Socioeconomic gradients were evident through both educational attainment (declining across quartiles; *p* < 0.001) and income measures (PIR: 2.45 ± 1.51 in Q4 vs. 2.63 ± 1.64 in Q1; *p* < 0.001). Smoking rates (56.21% vs. 36.80%; *p* < 0.001) and BMI (29.15 ± 6.43 vs. 26.99 ± 5.66 kg/m^2^; *p* < 0.001) increased across quartiles.

**Figure 4:**
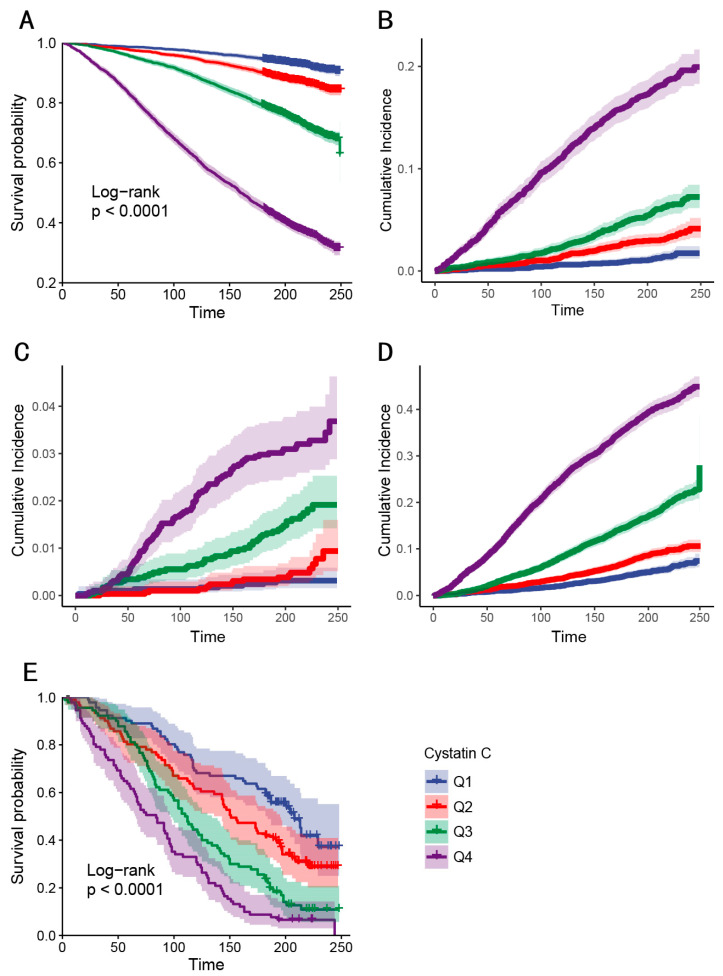
K-M analyses and cause-specific weighted cumulative incidence functions (CIFs) for mortality among the cystatin C four groups. (**A**) K-M analyses for all-cause mortality in all people. (**B**) Weighted cumulative incidence of cardiovascular mortality in all people. (**C**) Weighted cumulative incidence of cerebrovascular mortality in all people. (**D**) Weighted cumulative incidence of non-cardio-cerebrovascular mortality in all people. (**E**) K-M analyses for all-cause mortality in people with stroke. Q1–Q4, quartiles 1–4.

During follow-up, 3207 all-cause deaths (27.6%), 850 cardiovascular deaths (7.3%), 169 cerebrovascular deaths (1.5%), 2188 non-cardio-cerebrovascular deaths (18.9%), and 258 all-cause deaths in individuals with stroke (2.2%) were documented. The cerebrovascular mortality subgroup demonstrated comparable clinical profiles to cardiovascular decedents (n = 850) with similar hypertension prevalence (78.5% vs. 76.2%) and diabetes (34.8% vs. 31.9%), though they were significantly older (72.3 vs. 68.1 years, *p* < 0.001). Both groups shared elevated cystatin C levels versus non-cardio-cerebrovascular decedents (Table 4). Kaplan-Meier analyses demonstrated graded survival disparities, with Q4 showing the lowest survival probabilities for all-cause mortality in all people (log-rank *p* < 0.001; Figure 4A) and all-cause mortality in patients with stroke (log-rank *p* < 0.001; Figure 4E). For cause-specific mortality, the results of Gray’s test for the equality of weighted cumulative incidence function also confirmed that cystatin C was statistically significantly associated with cardiovascular (*p* < 0.001; Figure 4B), cerebrovascular mortality (*p* < 0.001; Figure 4C), and non-cardio-cerebrovascular (*p* < 0.001; Figure 4D) in all people. Additionally, time-stratified survival analyses comparing Q2 and Q3 mortality risks before and after the crossing point at 67 months revealed no significant difference during the early phase (<67 months; *p* = 0.326) but significantly higher mortality in Q3 during the late phase (≥67 months; *p* = 0.0003) (Supplementary Table S3).

Cause-specific Cox regression revealed dose-dependent mortality risks. Each 1 mg/L cystatin C increase predicted 48% higher all-cause mortality (HR = 1.48, 95% CI: 1.39–1.57; *p* < 0.001), with Q4 exhibiting 2.31-fold risk versus Q1 (*p* for trend < 0.001; Table 5). Cardiovascular mortality exhibited similar trends, with a 2.14-fold risk increase in Q4 (HR = 2.14, 1.33–3.44; *p* = 0.002). For cerebrovascular mortality, no significant association was observed in fully adjusted models (Q4 HR = 1.80, 95% CI: 0.65–5.01; *p* = 0.258). Notably, non-cardio-cerebrovascular mortality showed a robust graded relationship (Q4 HR = 2.43, 95% CI: 1.95–3.02; *p* < 0.001). Among patients with stroke, all-cause mortality risk increased with higher cystatin C levels (Q4 HR = 1.35, 0.73–2.51; *p* = 0.338), although the trend was not statistically significant (*p* for trend = 0.134). Model fitness was assessed using AIC and BIC criteria, and the multivariable-adjusted models demonstrated superior fit compared to unadjusted models (Supplementary Table S8).

To align with clinical practice, we applied the established reference range for cystatin C (0.51–0.98 mg/L; Finney et al. [18]) and categorized participants into normal (≤0.98 mg/L) and elevated (>0.98 mg/L) groups. Elevated cystatin C predicted significantly higher risks of all-cause mortality (HR = 1.79, 95% CI: 1.61–1.98; *p* < 0.001), cardiovascular mortality (HR = 1.94, 95% CI: 1.59–2.36; *p* < 0.001), and non-cardio-cerebrovascular mortality (HR = 1.77, 95% CI: 1.55–2.01; *p* < 0.001) in fully adjusted models. However, associations with cerebrovascular mortality (HR = 1.14, 95% CI: 0.67–1.92; *p* = 0.632) and all-cause mortality in stroke patients (HR = 1.25, 95% CI: 0.87–1.79; *p* = 0.228) were not statistically significant (Supplementary Table S4).

To address sparse events in cardiovascular and cerebrovascular mortality endpoints, Firth’s penalized Cox regression was utilized, yielding cause-specific HR estimates consistent with cause-specific Cox models (Supplementary Table S5). Optimal Youden’s index thresholds were all-cause mortality = 0.909 mg/L (HR = 1.77, 95% CI: 1.61–1.94), cardiovascular mortality = 0.868 mg/L (HR = 1.62, 95% CI: 1.35–1.95), and cerebrovascular mortality = 0.817 mg/L (HR = 1.44, 95% CI: 0.90–2.31; Supplementary Table S6). Supplementary Figure S1B confirms cystatin C’s discriminative capacity for all-cause mortality (AUC = 0.810). At the 0.909 mg/L cutoff, sensitivity reached 79.1% with a specificity of 72.9%. For cardiovascular mortality (Supplementary Figure S1C), the 0.868 mg/L cutoff achieved sensitivity = 73.2% and specificity = 78.4% with AUC = 0.827. Cerebrovascular mortality (Supplementary Figure S1D) showed AUC = 0.724 with sensitivity = 64.0% at 0.817 mg/L. Non-cardio-cerebrovascular mortality (Supplementary Figure S1E) demonstrated AUC = 0.806 (95% CI: 0.68–0.72) with sensitivity = 83.0% and specificity = 72.8% at 0.949 mg/L. For all-cause mortality in stroke patients (Supplementary Figure S1F), the optimal cutoff was 0.996 mg/L (AUC = 0.579, sensitivity = 57.9%, and specificity = 76.5%,).

**Figure 5:**
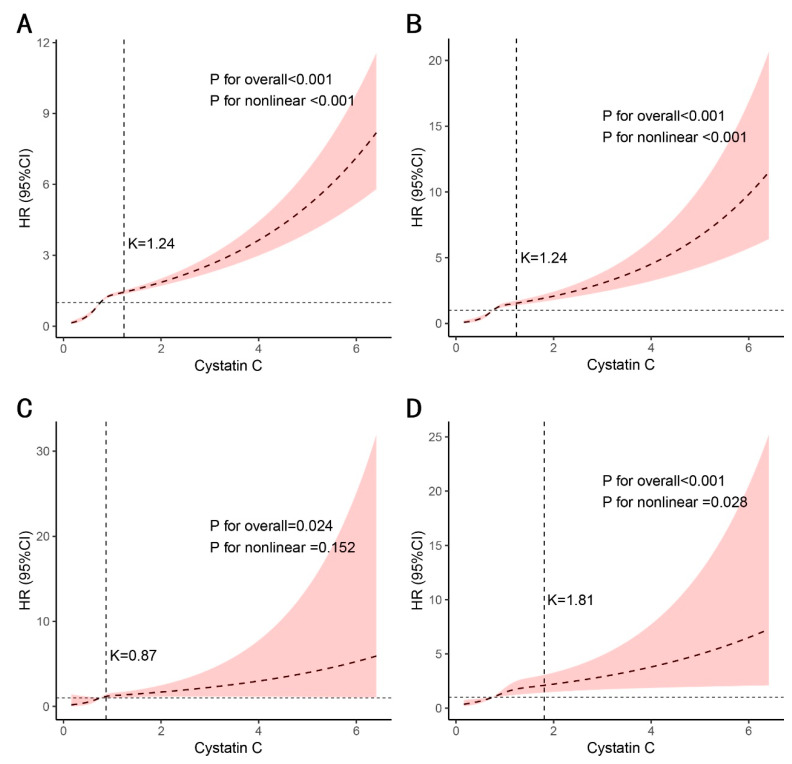
RCS analyses between serum cystatin C levels and mortality outcomes. Association of the cystatin C with all-cause mortality (**A**), cardiovascular mortality (**B**), and cerebrovascular mortality (**C**) in all people. (**D**) Association of cystatin C with all-cause mortality in people with stroke. Adjusted for age, sex, and race. The solid line and purple area represent the estimated values and their corresponding 95% CIs, respectively.

RCS analyses identified nonlinear thresholds for all-cause mortality (inflection point: 1.24 mg/L; *p* for nonlinear < 0.001; Figure 5A, Table 6) and cardiovascular mortality (1.24 mg/L; *p* for nonlinear < 0.001; Figure 5B, Table 6), with substantially steeper risk gradients below these thresholds (HR = 6.73 and 10.60 per 0.1 mg/L increase, respectively; both *p* < 0.001). For cerebrovascular mortality, no significant nonlinear pattern was observed (*p* for nonlinear = 0.152; Figure 5C, Table 6), and piecewise regression indicated equivalent model fit between threshold and linear models (log-likelihood ratio *p* = 0.064), suggesting a potential linear association (HR = 1.43 per 1 mg/L increase, *p* = 0.008). All-cause mortality in stroke patients exhibited a distinct inflection point at 1.81 mg/L (*p* for nonlinear < 0.001; Figure 5D, Table 6), with significant risk elevation below this threshold.

**Figure 6:**
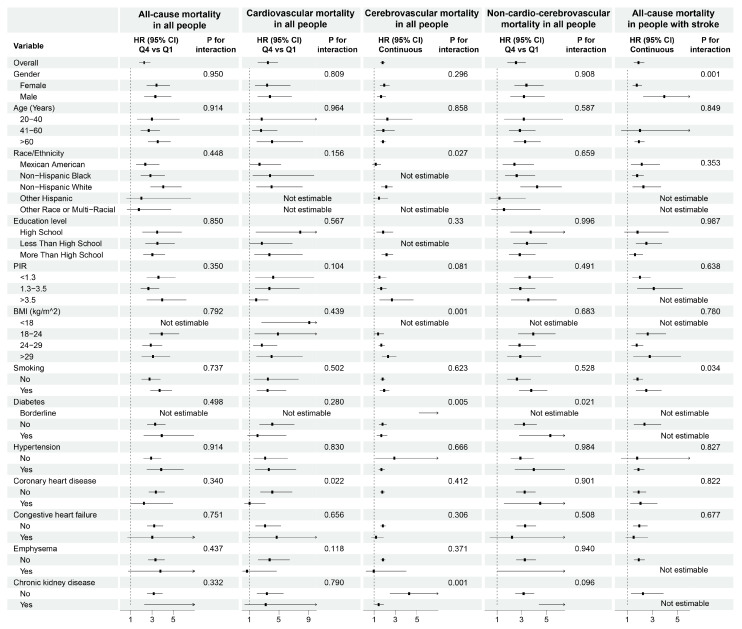
Subgroup analyses of the association between cystatin C and mortality. Adjusted for age, gender, race, educational level, family income-to-poverty ratio, BMI, hypertension, diabetes, chronic kidney disease, and smoking status. BMI, body mass index. PIR, Family income-to-poverty ratio.

Subgroup analyses consistently demonstrated a positive association between elevated cystatin C levels and mortality risks across diverse populations (Figure 6). For all-cause mortality (Figure 6), while variables such as age, gender, education level, smoking, hypertension, diabetes, and cardiovascular comorbidities independently predicted mortality (*p* < 0.05 for main effects), only diabetes (*p* for interaction = 0.009), CHF (*p* for interaction = 0.02), and CKD (*p* for interaction < 0.001) significantly modified the cystatin C–mortality relationship. Specifically, the association was attenuated in individuals with diabetes (HR = 1.53 vs. 1.84 in non-diabetes) or CHF (HR = 1.38 vs. 1.82 in non-CHF), whereas non-CKD subgroups exhibited paradoxically stronger associations (HR = 4.64 vs. 1.35 in CKD). In cardiovascular mortality analyses (Figure 6), interaction tests identified significant effect modifications for age (*p* for interaction = 0.005), diabetes (*p* for interaction = 0.0037), and CKD (*p* for interaction < 0.001). For cerebrovascular mortality (Figure 6), non-Hispanic White individuals (HR = 2.62, 1.54–4.47; *p* for interaction = 0.032), participants with BMI ≥ 29 kg/m^2^ (HR = 2.49, 1.63–3.81; *p* for interaction = 0.002), and non-CKD (HR = 4.35, 2.45–7.71; *p* for interaction = 0.001) significantly modified the association between cystatin C and mortality risk. Ethnic-specific distributions for cerebrovascular mortality are detailed in Supplementary Table S7. The small sample sizes in minority groups (Other Hispanic: n = 7; Other/Multiracial: n = 3) result in unstable estimates, as seen in preliminary analyses (e.g., Other Hispanic: HR = 0.00, 0.00–2.14), necessitating extreme caution in interpreting ethnic subgroup results. All-cause mortality analysis in patients with stroke (Figure 6) revealed amplified risks in males (HR = 4.63, 2.73–7.84; *p* for interaction = 0.003), multiracial/other ethnic groups (HR = 10.22, 2.12–49.35; *p* for interaction = 0.01), and subgroups with more than a high school education (HR = 6.96, 3.80–12.73; *p* for interaction = 0.047). These findings emphasize that while traditional risk factors independently contribute to mortality, cystatin C’s prognostic utility is dynamically influenced by specific comorbidities, necessitating contextual interpretation in clinical risk stratification.

## 4. Discussion

This study elucidates the independent prognostic utility of serum cystatin C in predicting stroke morbidity and cardio-cerebrovascular mortality. It reveals a nonlinear association with stroke morbidity, characterized by a critical threshold of 1.24 mg/L. Notably, this relationship persisted after comprehensive adjustment for potential confounding factors, including socioeconomic determinants and comorbid conditions, suggesting that cystatin C may reflect vascular injury pathways distinct from traditional risk mediators. Mortality analyses demonstrated endpoint-specific dynamics: all-cause and cardiovascular risks plateaued above 1.24 mg/L, while all-cause mortality in patients with stroke exhibited a distinct threshold effect at 1.81 mg/L with significant risk elevation below this cutoff. These findings challenge linear risk assumptions and highlight the biomarker’s potential to stratify heterogeneous risk trajectories, particularly in individuals without advanced comorbidities. By integrating population-level thresholds with clinical endpoints, our results provide actionable insights for refining risk stratification frameworks and tailoring preventive interventions to specific risk gradients.

### 4.1. Association Between Cystatin C and Stroke Morbidity

Our findings demonstrate robust evidence supporting the role of serum cystatin C as an independent predictor of stroke morbidity after full adjustment for comorbidities (Model 3 OR = 1.27, 95% CI: 1.02–1.58), in line with previous observational and genetic studies. Meta-analytic data consistently demonstrate an association between elevated cystatin C levels and an increased incidence of ischemic stroke, particularly in acute-phase and subclinical cerebral infarction subgroups [20]. A national cohort analysis utilizing the China Health and Retirement Longitudinal Study (CHARLS) database revealed that participants in the highest cystatin C quartile exhibited a 38% higher risk of stroke morbidity (adjusted OR: 1.38, 95% CI: 1.046–1.825), and Mendelian randomization studies confirmed a causal relationship, demonstrating that genetically predicted cystatin C elevation is associated with a 11% higher stroke risk per standard deviation increase (adjusted OR: 1.128, 95% CI: 1.058–1.203) [21].

However, it is important to acknowledge the existence of discrepancies in the literature. The Tromsø Study, for instance, failed to demonstrate an independent association between cystatin C and incident ischemic stroke after multivariable adjustment, although it did identify cystatin C as a mortality risk factor specifically in women (adjusted HR: 1.38 for the highest quartile) [14]. Similarly, a threshold analysis by Aguilar MI et al. revealed an increased ischemic stroke risk below 1.28 mg/L, but no association with hemorrhagic stroke [22]. These discrepancies likely reflect population heterogeneity, as evidenced by racial and gender-specific risk gradients observed in the Reasons for Geographic And Racial Differences in Stroke (REGARDS) cohort [23].

Our findings extend current knowledge by demonstrating a nonlinear relationship with an inflection point at 1.24 mg/L (*p* for nonlinearity < 0.001). Subthreshold cystatin C levels (<1.24 mg/L) are associated with an amplified risk of stroke, while suprathreshold concentrations exhibit attenuated effects. Notably, this nonlinear pattern remained significant even after extensive adjustments for potential confounding factors, including socioeconomic determinants and comorbid conditions, thereby strengthening the evidence for cystatin C’s independent prognostic value. Crucially, while the interaction tests for comorbidities showed no statistically significant effect modification (*p* for interaction > 0.05), point estimates revealed clinically meaningful gradients. Participants without CKD exhibited a substantially higher risk. The Youden’s index-derived cut-off of 0.885 mg/L for stroke morbidity corroborated our quartile-based risk stratification, where Q4 started at 0.883 mg/L, supporting the clinical relevance of this threshold. The RCS-identified threshold at 1.24 mg/L fundamentally redefines clinical interpretation compared to linear models. Where logistic regression suggested uniform risk elevation, RCS revealed that cystatin C < 1.24 mg/L carries exponentially increasing stroke risk per unit increment—a finding with profound implications for early intervention. This supports cystatin C as a screening biomarker for incipient vascular injury rather than merely a risk marker in established disease.

These results underscore the importance of considering nonlinear relationships in biomarker research and highlight the need for further investigation into the biological mechanisms underlying the differential risk associations across varying cystatin C concentrations. Additionally, the consistency of our findings with the clinical reference range established by Finney et al. [18] adds further validity to the proposed threshold of 0.98 mg/L for elevated cystatin C, which was associated with 2.03-fold significantly higher odds of stroke morbidity in fully adjusted models (Supplementary Table S1). This alignment with established clinical benchmarks strengthens the translational potential of our findings and facilitates their integration into existing clinical frameworks for stroke risk assessment.

### 4.2. Association Between Cystatin C and Mortality

The observed associations between elevated cystatin C levels and the risks of all-cause and cardiovascular mortality are consistent with established epidemiological evidence. Pioneering work by Shlipak MG et al. initially demonstrated cystatin C’s prognostic value for all-cause mortality in older adults, independent of renal function [9]. Subsequently, mechanistic studies further elucidated cystatin C’s involvement in vascular pathophysiology, with elevated levels promoting oxidative stress and arterial calcification—processes directly linked to cardiovascular event risk [24]. In high-risk populations, cystatin C consistently predicts adverse outcomes, including mortality, heart failure, and recurrent cardiovascular events, even after adjustment for traditional risk factors [25,26,27]. Extensive meta-analyses corroborate these findings, establishing cystatin C as a reliable biomarker for cardiovascular risk stratification across diverse cohorts [28,29,30,31]. Our study extends this evidence by elucidating nonlinear dose–response relationships, identifying a shared inflection point at 1.24 mg/L for both all-cause and cardiovascular mortality. Below this threshold, each 0.1 mg/L increment in cystatin C corresponded to a 6.73-fold mortality risk elevation (β = 6.727, *p* < 0.001), whereas elevated levels exhibited attenuated but persistent associations (β = 1.333, *p* < 0.001). This threshold-driven model enhances clinical interpretability, providing actionable cutoffs for risk stratification while reconciling prior inconsistencies in linear risk assumptions. By quantifying cystatin C’s differential predictive utility across concentration gradients, our findings refine its role in precision prevention strategies, particularly in populations where traditional risk factors fail to adequately capture residual risk.

Emerging evidence suggests cystatin C’s prognostic significance in cerebrovascular mortality. A meta-analysis conducted by Hao C and Chen S [11] demonstrated significantly elevated serum cystatin C concentrations in non-survivors of acute ischemic stroke compared to survivors, indicating its mortality risk association [11]. These findings are consistent with cohort studies reporting elevated cystatin C levels in acute ischemic stroke non-survivors [12,13], corroborated by our analysis showing a 2.01-fold cerebrovascular mortality risk (HR = 2.01, 95% CI: 1.43–2.81; *p* < 0.001). Although a potential linear trend between cystatin C and cerebrovascular mortality was observed, statistical non-significance (*p* for trend = 0.068) may reflect limited power, heterogeneous follow-up durations, or residual confounding by renal function and comorbidities. Consequently, further validation in larger cohorts is warranted.

Furthermore, cystatin C-estimated glomerular filtration rate (eGFR) outperforms creatinine-based eGFR in predicting all-cause mortality in patients with ischemic stroke [32], consistent with our findings of elevated all-cause mortality in stroke patients with higher cystatin C levels. A nonlinear relationship was observed with a specific threshold (1.81 mg/L), where statistical significance was only evident below this threshold. This suggests that the association between cystatin C and all-cause mortality in patients with stroke may follow a dose-dependent pattern, with no additional mortality risk observed beyond the threshold. This pattern may arise from ceiling effects in high-risk populations, where extreme baseline mortality attenuates incremental biomarker effects. Significant interaction effects across subgroups (*p* for interaction < 0.05) further emphasize the need for multifactorial risk models integrating cystatin C with clinical parameters to optimize prognostic accuracy. Notably, our analysis of non-cardio-cerebrovascular mortality revealed a robust graded relationship (HR = 1.77, 95% CI: 1.55–2.01; *p* < 0.001 for the highest cystatin C quartile), underscoring cystatin C’s systemic impact beyond cardiovascular pathways. This finding aligns with prior studies implicating cystatin C in inflammatory and oxidative stress mechanisms that drive multi-organ dysfunction.

Finally, the application of Firth’s penalized Cox regression to address sparse event counts in cardiovascular and cerebrovascular mortality endpoints validated our primary results, demonstrating consistent hazard ratios across methods. This methodological rigor strengthens the reliability of our observed associations and supports the clinical relevance of cystatin C as a mortality risk predictor in diverse populations.

### 4.3. Potential Mechanisms Linking Serum Cystatin C Levels to Stroke Morbidity and Cause-Specific Cardio-Cerebrovascular Mortality

Serum cystatin C, an endogenous cysteine protease inhibitor, regulates vascular proteolytic balance by suppressing cysteine cathepsin activity, thereby modulating atherosclerotic plaque formation and stability [15]. Synthesized constitutively by nucleated cells, its low molecular weight enables unrestricted glomerular filtration, establishing it as a superior renal function biomarker compared to creatinine [33,34]. Beyond its role in renal clearance, cystatin C exerts multifactorial influences on cerebrovascular and cardiovascular outcomes. Mechanistically, cystatin C contributes to atherosclerosis by disrupting the balance between vascular proteases and antiproteases (e.g., inhibiting cysteine cathepsin activity), promoting plaque formation and instability, thereby increasing stroke risk [17,34]. Elevated cystatin C levels are indicative of impaired renal function, and chronic kidney dysfunction is associated with oxidative stress and systemic inflammation, which accelerate atherosclerosis [35]. These processes exacerbate vascular damage and increase the risk of stroke recurrence [36,37]. At the neurological level, cystatin C directly damages neural cells by inducing inflammatory responses and neuronal apoptosis, while impairing cerebral amyloid clearance, leading to cognitive dysfunction [38,39]. Its association with cerebral microbleeds may further impair cognitive function by disrupting white matter integrity [40].

Additionally, cystatin C activates inflammatory pathways (e.g., NF-κB) and promotes the release of pro-inflammatory cytokines (e.g., IL-6, CRP), establishing a vicious cycle of post-stroke neuroinflammation and delayed neuronal death [41], significantly increasing all-cause mortality. Our mortality analyses demonstrated that cystatin C’s association with non-cardio-cerebrovascular mortality (HR = 1.77, 95% CI: 1.55–2.01; *p* < 0.001) is comparable in magnitude to its links with cardiovascular outcomes, underscoring its role in systemic inflammation and multi-organ damage.

Furthermore, cystatin C also contributes to cardiovascular mortality through mechanisms extending beyond cerebrovascular effects, including direct roles in coronary atherosclerosis and myocardial injury [25,26,27,28,29,30,31,32]. The shared inflection point of 1.24 mg/L for all-cause and cardiovascular mortality in our study suggests that the biomarker’s impact on vascular health may be mechanistically linked across different endpoints. This threshold may represent a critical transition point where compensatory physiological mechanisms fail, leading to an accelerated risk of adverse events.

Synthesizing the evidence from above, cystatin C serves as a pivotal biomarker for both stroke and cardiovascular mortality, driven by interconnected pathways such as atherosclerosis, inflammation, oxidative stress, and cardiac damage. Its dual impact on cerebrovascular and cardiovascular systems highlights the potential for early monitoring and targeted interventions to enhance clinical outcomes.

### 4.4. Clinical Implications Derived from Current Evidence

In summary, the clinical application of cystatin C can be extended to optimize risk stratification tools. For instance, incorporating thresholds (1.24 mg/L and 1.81 mg/L) could guide personalized management strategies: intensifying lifestyle interventions for low-risk populations (cystatin C < 1.24 mg/L) and initiating pharmacological interventions or close monitoring for moderate- to high-risk groups (cystatin C ≥ 1.24 mg/L). Mechanistically, cystatin C’s dual role as a biomarker and therapeutic target—via modulation of vascular proteolytic balance and inflammatory cascades—warrants exploration in randomized controlled trials (RCTs) to assess causality between targeted cystatin C reduction and improved outcomes. Public health initiatives should prioritize cystatin C integration into routine cardio-cerebrovascular screening protocols, paralleling lipid profile assessments, to optimize early detection. The consistency of our findings with established clinical benchmarks, such as the reference range established by Finney et al. [18] (0.51–0.98 mg/L), strengthens the translational potential of cystatin C. Elevated levels (>0.98 mg/L) were associated with significantly higher risks of all-cause mortality (HR = 1.79, 95% CI: 1.61–1.98) and cardiovascular mortality (HR = 1.94, 95% CI: 1.59–2.36), providing a direct link between existing clinical standards and our observed risk gradients.

Future investigations should prioritize (1) mechanistic studies elucidating cystatin C’s endothelial dysfunction and oxidative stress pathways; (2) intervention trials evaluating dietary, pharmacological, or exercise-based strategies; and (3) population-specific threshold calibration. This translational roadmap positions cystatin C as a precision medicine tool, bridging biomarker discovery to individualized prevention.

### 4.5. Limitation

This study has several methodological limitations. First, disease ascertainment relied on self-reported diagnoses, which are susceptible to recall bias and potential misclassification. Second, the NHANES database does not differentiate between the two primary stroke types (ischemic or hemorrhagic) or stroke subtypes, which may obscure etiology-specific cystatin C associations. Third, the observational cross-sectional design for the stroke morbidity analysis precludes causal inference. While we adjusted for multiple confounders, the possibility of residual confounding from unmeasured variables, such as subclinical inflammation or detailed dietary patterns, cannot be ruled out. Similarly, for the mortality analyses, although we used a longitudinal cohort design, the reliance on baseline cystatin C measurements limits our ability to capture temporal changes in biomarker levels that might influence mortality risk. Fourth, sparse cerebrovascular mortality events (*n* = 169) limit subgroup precision and statistical power, which reduced the reliability of estimates and contributed to non-significant trends in some analyses. These findings should be interpreted cautiously. Fifth, although we rigorously adjusted for established confounders, including renal function, cardiovascular comorbidities, and socioeconomic factors in multivariable models, residual confounding from unmeasured variables, such as genetic predispositions, dietary patterns, and subclinical inflammation, may still influence interpretations of ‘independent’ associations. This limitation underscores that while cystatin C demonstrates significant predictive value beyond traditional risk factors, its complete independence from all biological pathways cannot be definitively established. Finally, the generalizability of our findings may be affected by the exclusion of participants with missing data, although we conducted extensive sensitivity analyses to mitigate this concern. Additionally, the application of Firth’s penalized Cox regression for endpoints with sparse events introduced methodological complexity, and while it addressed small-sample bias, it requires validation in larger cohorts. Moreover, while Supplementary Table S7 provides full ethnic distribution data, the limited cerebrovascular deaths in minority groups (Other Hispanic: n = 7; Other/Multiracial: n = 3) constrain detailed ethnic-specific inferences. Despite these limitations, our study provides valuable insights into the nonlinear associations between cystatin C and cardio-cerebrovascular outcomes. Future research should address these limitations through prospective cohort studies with neuroimaging-confirmed stroke subtypes, repeated biomarker measurements, and detailed assessment of potential confounders to further validate the observed associations and clarify the causal pathways.

## 5. Conclusions

This study identifies serum cystatin C as an independent predictor after full adjustment of stroke morbidity, all-cause and cardio-cerebrovascular mortality, with distinct nonlinear thresholds at 1.24 mg/L for stroke morbidity, all-cause mortality, and cardiovascular mortality, and at 1.81 mg/L for all-cause mortality in stroke patients. Below the 1.24 mg/L threshold, each 0.1 mg/L increase in cystatin C conferred 13.84-fold higher stroke risk (95% CI: 7.11–27.04), demonstrating exponential risk elevation in the subthreshold range. Clinically meaningful risk gradients were observed in subgroups without advanced comorbidities, though formal interaction tests were non-significant. Critically, cystatin C exhibited pan-systemic prognostic value, evidenced by robust associations with non-cardio-cerebrovascular mortality (Q4 cause-specific HR = 2.43, 95% CI: 1.95–3.02). These findings position cystatin C as a dual-purpose biomarker for early vascular injury detection in subclinical populations and integrated mortality risk stratification. Future research should validate these thresholds in prospective, neuroimaging-confirmed cohorts and explore interventions targeting cystatin C pathways to optimize prevention strategies.

## Figures and Tables

**Table 1:** Weighted comparison in basic characteristics.

Characteristic	Overall N = 11,610	Non-Stroke N = 11,248	Stroke N = 362	Weighted *p*-Value
Age (Years), Mean ± SD	49.73 ± 18.76	49.15 ± 18.60	67.57 ± 14.37	<0.001
Gender, %				0.165
Female	51.69	51.80	48.07	
Male	48.31	48.20	51.93	
Race/Ethnicity, %				0.031
Mexican American	22.19	22.32	17.96	
Non-Hispanic Black	17.92	17.85	19.89	
Non-Hispanic White	51.90	51.75	56.63	
Other Hispanic	4.52	4.60	2.21	
Other Race or Multi-Racial	3.47	3.48	3.31	
PIR, Mean ± SD	2.65 ± 1.61	2.67 ± 1.61	2.20 ± 1.44	<0.001
Education level, %				<0.001
High School	24.07	24.11	22.93	
Less Than High School	31.22	30.76	45.58	
More Than High School	44.70	45.13	31.49	
BMI (kg/m^2^), Mean ± SD	28.36 ± 6.19	28.33 ± 6.19	29.32 ± 6.07	<0.001
Smoking, %	49.03	48.82	55.52	0.012
Diabetes, %				<0.001
Borderline	1.35	1.30	3.04	
No	89.10	89.6	73.76	
Yes	9.54	9.10	23.20	
Hypertension, %	31.66	30.38	71.55	<0.001
Coronary heart disease, %	4.40	3.95	18.51	<0.001
Congestive heart failure, %	2.94	2.52	15.75	<0.001
Emphysema, %	1.89	1.73	6.63	<0.001
Chronic kidney disease, %	2.64	2.41	9.67	<0.001
Cystatin C (mg/L), Mean ± SD	0.82 ± 0.36	0.81 ± 0.35	1.09 ± 0.55	<0.001
Cystatin C quartiles, %				<0.001
Q1	25.11	25.76	4.70	
Q2	25.05	25.43	13.26	
Q3	24.92	25.09	19.61	
Q4	24.93	23.72	62.43	
All-cause mortality, %	27.65	26.13	74.86	<0.001

Mean ± SD for continuous variables: *p* value was calculated by weighted logistic regression model. % for categorical variables: *p* value was calculated by weighted chi-square test. BMI, body mass index. PIR, Family income-to-poverty ratio.

**Table 2:** Association between cystatin C and stroke morbidity.

	Model 1		Model 2		Model 3	
	OR [95% CI]	*p*-Value	OR [95% CI]	*p*-Value	OR [95% CI]	*p*-Value
Continuous	2.28 (1.66, 3.12)	<0.001	1.65 (1.39, 1.95)	<0.001	1.27 (1.02, 1.58)	0.024
Q1	Reference	-	Reference	-	Reference	-
Q2	2.51 (1.20, 5.27)	0.016	2.28 (1.07, 4.88)	0.034	1.99 (0.83, 4.77)	0.116
Q3	3.99 (2.01, 7.95)	<0.001	3.01 (1.47, 6.16)	0.004	2.46 (1.13, 5.35)	0.024
Q4	15.94 (8.39, 30.27)	<0.001	7.04 (3.47, 14.29)	<0.001	4.59 (2.10, 10.06)	<0.001
*p* for trend	<0.001		<0.001		<0.001	

Model 1: no covariates were adjusted. Model 2: age, gender, and race were adjusted. Model 3: age, gender, race, educational level, family income-to-poverty ratio, BMI, hypertension, diabetes, chronic kidney disease, and smoking status.

**Table 3:** Threshold effect analysis of cystatin C on stroke morbidity in the NHANES cohort.

Cystatin C	OR (95% CI), *p* Value
Model 1 A straight-line effect	1.65 (1.39, 1.95), <0.001
Model 2	
Inflection points (K)	1.24
<K segment effect	13.84 (7.11–27.04), <0.001
>K segment effect	1.02 (0.78–1.26), 0.872
Logarithmic likelihood ratio test	<0.001
95%CI of the Cut point	1.20, 1.68

Adjusted for age, gender, and race/ethnicity.

**Table 4:** Baseline demographic and clinical data of cystatin C four quantiles.

	Q1	Q2	Q3	Q4	Overall N = 11,598	Weighted *p*-Value
	<0.163	0.163–0.661	0.661–0.883	>0.883		
	N = 2910	N = 2907	N = 2892	N = 2889		
Cystatin C, Mean ± SD	0.59 ± 0.06	0.71 ± 0.03	0.81 ± 0.04	1.16 ± 0.57	0.82 ± 0.36	<0.001
Gender, %						<0.001
Female	68.04	48.99	42.46	47.18	51.69	
Male	31.96	51.01	57.54	52.82	48.31	
Age (Years), Mean ± SD	38.05 ± 13.00	43.81 ± 15.45	51.09 ± 17.40	66.09 ± 16.08	49.73 ± 18.77	<0.001
Race/Ethnicity, %						<0.001
Mexican American	30.48	24.05	18.88	15.33	22.20	
Non-Hispanic Black	23.40	19.47	15.25	13.53	17.93	
Non-Hispanic White	36.05	48.57	58.06	64.97	51.88	
Other Hispanic	5.40	4.37	4.94	3.39	4.53	
Other Race or Multi-Racial	4.67	3.54	2.87	2.77	3.47	
Education level, %						<0.001
High School	21.92	23.8	24.86	25.72	24.07	
Less Than High School	28.73	27.83	30.46	37.97	31.24	
More Than High School	49.35	48.37	44.67	36.31	44.69	
PIR, Mean ± SD	2.63 ± 1.64	2.80 ± 1.66	2.73 ± 1.61	2.45 ± 1.51	2.65 ± 1.61	<0.001
BMI (kg/m^2^), Mean ± SD	26.99 ± 5.66	28.27 ± 6.06	29.03 ± 6.34	29.15 ± 6.43	28.36 ± 6.19	<0.001
Smoking, %	36.80	48.13	54.91	56.21	48.99	<0.001
Diabetes, %						<0.001
Borderline	0.79	0.96	1.18	2.46	1.35	
No	93.02	93.22	89.28	80.86	89.11	
Yes	6.19	5.81	9.54	16.68	9.54	
Hypertension, %	16.43	24.01	32.43	53.93	31.66	<0.001
Coronary heart disease, %	0.89	2.13	3.94	10.66	4.40	<0.001
Congestive heart failure, %	0.38	0.86	2.18	8.38	2.94	<0.001
Emphysema, %	0.62	0.83	2.04	4.02	1.87	<0.001
Chronic kidney disease, %	1.03	1.20	1.59	6.72	2.63	<0.001

Mean ± SD for continuous variables: *p* value was calculated by weighted logistic regression model. % for categorical variables: *p* value was calculated by weighted chi-square test. BMI, body mass index. PIR, Family income-to-poverty ratio.

**Table 5:** Cause-specific Cox regression models for the association between cystatin C and mortality.

	Model 1		Model 2		Model 3		Number of Deaths
	HR [95% CI]	*p*-Value	HR [95% CI]	*p*-Value	HR [95% CI]	*p*-Value	
All-cause mortality in all people							
Continuous	1.99 (1.93, 2.05)	<0.05	1.64 (1.57, 1.72)	<0.001	1.48 (1.39, 1.57)	<0.001	3207
Q1	Reference	-	Reference	-	Reference	-	213
Q2	1.91 (1.56, 2.35)	<0.001	1.26 (1.02, 1.56)	0.03	1.27 (1.02, 1.58)	0.035	385
Q3	3.79 (3.13, 4.59)	<0.001	1.58 (1.32, 1.9)	<0.001	1.48 (1.18, 1.86)	<0.001	787
Q4	13.79 (11.34, 16.77)	<0.001	2.62 (2.17, 3.15)	<0.001	2.31 (1.87, 2.86)	<0.001	1822
*p* for trend	<0.001		<0.001		<0.001		
Cardiovascular mortality in all people							
Continuous	2.06 (1.96, 2.17)	<0.001	1.78 (1.65, 1.92)	<0.001	1.51 (1.36, 1.68)	<0.001	850
Q1	Reference	-	Reference	-	Reference	-	39
Q2	2.59 (1.50, 4.49)	<0.001	1.57 (0.91, 2.71)	0.108	1.42 (0.83, 2.44)	0.203	97
Q3	4.53 (2.94, 6.97)	<0.001	1.60 (1.05, 2.43)	0.03	1.34 (0.86, 2.1)	0.195	180
Q4	21.47 (13.15, 35.04)	<0.001	3.11 (1.98, 4.88)	<0.001	2.14 (1.33, 3.44)	0.002	534
*p* for trend	<0.001		<0.001		<0.001		
Cerebrovascular mortality in all people							
Continuous	1.96 (1.72, 2.24)	<0.001	1.53 (1.22, 1.92)	<0.001	1.38 (1.02, 1.87)	0.034	169
Q1	Reference	-	Reference	-	Reference	-	9
Q2	1.64 (0.59, 4.54)	0.338	0.95 (0.36, 2.52)	0.916	1.27 (0.44, 3.64)	0.662	17
Q3	4.22 (1.61, 11.02)	0.003	1.33 (0.53, 3.38)	0.544	1.32 (0.47, 3.69)	0.602	49
Q4	13.45 (5.23, 34.58)	<0.001	1.58 (0.63, 3.94)	0.328	1.80 (0.65, 5.01)	0.258	94
*p* for trend	<0.001		0.112		0.125		
Non-cardio-cerebrovascular mortality in all people							
Continuous	1.95 (1.88, 2.03)	<0.001	1.59 (1.5, 1.69)	<0.001	1.47 (1.37, 1.59)	<0.001	2188
Q1	Reference	-	Reference	-	Reference	-	165
Q2	1.77 (1.40, 2.23)	<0.001	1.21 (0.95, 1.54)	0.114	1.23 (0.97, 1.57)	0.092	271
Q3	3.59 (2.92, 4.42)	<0.001	1.62 (1.33, 1.97)	<0.001	1.60 (1.29, 1.97)	<0.001	558
Q4	11.97 (9.89, 14.48)	<0.001	2.57 (2.1, 3.16)	<0.001	2.43 (1.95, 3.02)	<0.001	1194
*p* for trend	<0.001		<0.001		<0.001		
All-cause mortality in people with stroke							
Continuous	1.79 (1.57, 2.03)	<0.001	1.67 (1.42, 1.97)	<0.001	1.44 (1.16, 1.79)	0.001	258
Q1	Reference	-	Reference	-	Reference	-	45
Q2	1.51 (0.95, 2.38)	0.079	0.81 (0.50, 1.3)	0.378	0.80 (0.49, 1.31)	0.383	58
Q3	2.59 (1.62, 4.14)	<0.001	1.05 (0.70, 1.56)	0.818	0.93 (0.59, 1.47)	0.756	74
Q4	4.14 (2.38, 7.19)	<0.001	1.47 (0.83, 2.58)	0.184	1.35 (0.73, 2.51)	0.338	81
*p* for trend	<0.001		0.023		0.134		

Model 1: no covariates were adjusted. Model 2: age, gender, and race were adjusted. Model 3: age, gender, race, educational level, family income-to-poverty ratio, BMI, hypertension, diabetes, chronic kidney disease, and smoking status.

**Table 6:** Threshold effect analysis of cystatin C on all-cause and cause-specific mortality in all people.

	HR (95% CI), *p* Value			
	All-Cause Mortality in All People	Cardiovascular Mortality in All People	Cerebrovascular Mortality in All People	All-Cause Mortality in People with Stroke
Model 1 A straight-line effect	1.64 (1.57, 1.72), <0.001	1.78 (1.65, 1.92), <0.001	1.53 (1.22, 1.92), <0.001	1.67 (1.42, 1.97), <0.001
Model 2				
Inflection points (K)	1.24	1.24	0.87	1.81
<K segment effect	6.73 (5.42–8.35), <0.001	10.60 (6.96–16.15), <0.001	12.68 (1.23–130.45), 0.033	3.59 (2.25–5.73), <0.001
>K segment effect	1.33 (1.24–1.43), <0.001	1.43 (1.28–1.60), <0.001	1.43 (1.10–1.86), 0.008	1.24 (0.94–1.64), 0.133
Logarithmic likelihood ratio test	<0.001	<0.001	0.064	<0.001
95% CI of the Cut point	1.37, 1.52	1.38, 1.73	0.99, 1.40	1.44, 3.06

Adjusted for age, sex, and race.

## Data Availability

Publicly available datasets were analyzed in this study. These data can be found at https://www.cdc.gov/nchs/nhanes/ (accessed on 1 August 2024).

## Supplementary Materials

The following supporting information can be downloaded at: https://www.mdpi.com/article/10.3390/healthcare13172137/s1, Table S1. Association between cystatin C and stroke morbidity. Table S2. Youden’s index-derived optimal cut-offs for stroke morbidity. Table S3. Time-stratified log-rank p-values for Q2 vs. Q3 comparison. Table S4. Cause-specific Cox regression models for the association between cystatin C and mortality. Table S5. Firth Cox regression models for the association between the cystatin C and mortality. Table S6. Mortality Risk Stratification by Youden’s Index-Derived Cystatin C Thresholds. Table S7. The mortalities in basic characteristics. Table S8. Model comparison using AIC and BIC criteria across different cystatin C specifications. Figure S1. The ROC curve utilized in assessing the predictive capability of Cystatin C for stroke morbidity and mortality. (A) ROC curve for stroke morbidity. (B) ROC curve for all-cause mortality in all people. (C) ROC curve for cardiovascular mortality in all people. (D) ROC curve for cerebrovascular mortality in all people. (E) ROC curve for non-cardio-cerebrovascular mortality in all people. (F) ROC curve for all-cause mortality in people with stroke.

## Abbreviations

The following abbreviations are used in this manuscript:

NHANES	National Health and Nutrition Examination Survey
CKD	Chronic kidney disease
CHD	Coronary heart disease
CHF	Congestive heart failure
DALYs	Disability-adjusted life years
GBD	Global Burden of Disease
ASIR	Age-standardized incidence rate
WHO	World Health Organization
GFR	Glomerular filtration rate
CDC	Centers for Disease Control and Prevention
NCHS	National Center for Health Statistics
CAPI	Computer-Assisted Personal Interviewing
NDI	National Death Index
ICD	International Classification of Diseases
SD	Standard deviations
RCS	Restricted cubic splines
HR	Hazard ratio
OR	Odds ratio
BMI	Body mass index
CHARLS	China Health and Retirement Longitudinal Study
REGARDS	Reasons for Geographic And Racial Differences in Stroke
RCTs	Randomized controlled trials
eGFR	estimated glomerular filtration rate
PIR	Family income-to-poverty ratio.
