# Supplementary material for: Association of Serum Cystatin C with Stroke Morbidity and All-Cause and Cardio-Cerebrovascular Mortality: Evidence from the NHANES
Source: Healthcare (Basel). 2025 Aug 27;13(17):2137. doi: 10.3390/healthcare13172137 (PMC12428255; doi:10.3390/healthcare13172137)
Supplement: Supplementary file 1 [file healthcare-13-02137-s001.zip › healthcare-3775205-supplementary.pdf]

Supplementary Materials

Table S1. Association between cystatin C and stroke morbidity.

	Model 1		Model 2		Model 3	
	**OR [95% CI]**	***P*-value**	**OR [95% CI]**	***P*-value**	**OR [95% CI]**	***P*-value**
Cystatin C ≤0.98	Reference	-	Reference	-	Reference	-
Cystatin C > 0.98	6.72(5.40,8.37)	<0.001	2.71(1.95,3.76)	<0.001	2.03(1.46,2.82)	<0.001

Model 1: no covariates were adjusted. Model 2: age, gender, and race were adjusted. Model 3: age, gender, race, educational level, family income-to-poverty ratio, BMI, hypertension, diabetes, chronic kidney disease, and smoking status.

Table S2. Youden's index-derived optimal cut-offs for stroke morbidity.

	Model 1		Model 2		Model 3	
	**OR [95% CI]**	***P*-value**	**OR [95% CI]**	***P*-value**	**OR [95% CI]**	***P*-value**
Cystatin C ≤0.885	Reference	-	Reference	-	Reference	-
Cystatin C > 0. 885	6.05(4.55,8.06)	<0.001	2.96(2.09,4.2)	<0.001	2.2(1.51,3.21)	<0.001

Model 1: no covariates were adjusted. Model 2: age, gender, and race were adjusted. Model 3: age, gender, race, educational level, family income-to-poverty ratio, BMI, hypertension, diabetes, chronic kidney disease, and smoking status. Cut-off selection used maximizing Youden's index.

Table S3. Time-stratified log-rank p-values for Q2 vs Q3 comparison.

Q3 vs Q2	LRPV	MTPV	TSPV
**Total**	0.002	0.140	**0.002**
**Time<67**	0.067	0.308	**0.326**
**Time≥67**	0.0003	0.704	**0.0003**

LRPV, p-value of the log-rank test; MTPV, p-value of the suggested stage-II test; TSPV, p-value of the two-stage test.

Table S4. Cause-specific Cox regression models for the association between cystatin C and mortality.

	Model 1		Model 2		Model 3		Number of deaths
	**HR [95% CI]**	***P*-value**	**HR [95% CI]**	***P*-value**	**HR [95% CI]**	***P*-value**	
**All-cause mortality in all people**							
Cystatin C ≤0.98	Reference	-	Reference	-	Reference	-	1701
Cystatin C > 0.98	7.47(6.63,8.42)	<0.001	2.01(1.83,2.21)	<0.001	1.79(1.61,1.98)	<0.001	1205
**Cardiovascular mortality in all people**							
Cystatin C ≤0.98	Reference	-	Reference	-	Reference	-	409
Cystatin C > 0.98	10.24(8.65,12.12)	<0.001	2.44(2.06,2.89)	<0.001	1.94(1.59,2.36)	<0.001	369
**Cerebrovascular mortality in all people**							
Cystatin C ≤0.98	Reference	-	Reference	-	Reference	-	95
Cystatin C > 0.98	6.26(4.26,9.2)	<0.001	1.24(0.79,1.95)	0.357	1.14(0.67,1.92)	0.632	57
**Non-cardio-cerebrovascular mortality in all people**							
Cystatin C ≤0.98	Reference	-	Reference	-	Reference	-	1197
Cystatin C > 0.98	6.68(5.84,7.65)	<0.001	1.92(1.71,2.15)	<0.001	1.77(1.55,2.01)	<0.001	779
**All-cause mortality in people with stroke**							
Cystatin C ≤0.98	Reference	-	Reference	-	Reference	-	97
Cystatin C > 0.98	2.56(1.85,3.54)	<0.001	1.39(1.04,1.84)	0.025	1.25(0.87,1.79)	0.228	135

Model 1: no covariates were adjusted. Model 2: age, gender, and race were adjusted. Model 3: age, gender, race, educational level, family income-to-poverty ratio, BMI, hypertension, diabetes, chronic kidney disease, and smoking status.

Table S5. Firth Cox regression models for the association between the cystatin C and mortality.

	Model 1		Model 2		Model 3		Number of deaths
	HR [95% CI]	*P*-value	HR [95% CI]	*P*-value	HR [95% CI]	*P*-value	
**All-cause mortality in all people**							
Continuous	1.99(1.93,2.04)	<0.001	1.64(1.57,1.72)	<0.001	1.48(1.39,1.57)	<0.001	3207
Q1	Reference	-	Reference	-	Reference	-	213
Q2	1.83(1.55,2.17)	<0.001	1.06(0.9,1.26)	0.483	1.09(0.91,1.31)	0.333	385
Q3	4.04(3.48,4.71)	<0.001	1.39(1.19,1.63)	<0.001	1.4(1.19,1.66)	<0.001	787
Q4	13.12(11.41,15.16)	<0.001	2.15(1.84,2.52)	<0.001	2.03(1.72,2.41)	<0.001	1822
P for trend	<0.001		<0.001		<0.001		
**Cardiovascular mortality in all people**							
Continuous	2.07(1.96,2.17)	<0.001	1.78(1.65,1.91)	<0.001	1.51(1.36,1.68)	<0.001	850
Q1	Reference	-	Reference	-	Reference	-	39
Q2	2.5(1.74,3.66)	<0.001	1.33(0.93,1.96)	0.123	1.32(0.91,1.97)	0.148	97
Q3	4.99(3.58,7.14)	<0.001	1.47(1.04,2.12)	0.026	1.36(0.95,1.99)	0.097	180
Q4	20.64(15.15,28.98)	<0.001	2.65(1.9,3.8)	<0.001	2.11(1.48,3.08)	<0.001	534
P for trend	<0.001		<0.001		<0.001		
**Cerebrovascular mortality in all people**							
Continuous	1.99(1.72,2.22)	<0.001	1.58(1.22,1.89)	0.002	1.41(1.03,1.84)	0.036	169
Q1	Reference	-	Reference	-	Reference	-	9
Q2	1.87(0.86,4.28)	0.113	0.98(0.45,2.26)	0.953	1.03(0.44,2.65)	0.94	17
Q3	5.68(2.97,12.12)	<0.001	1.6(0.82,3.48)	0.178	1.6(0.76,3.87)	0.227	49
Q4	15.12(8.18,31.59)	<0.001	1.77(0.9,3.86)	0.101	1.73(0.81,4.19)	0.163	94
P for trend	<0.001		0.028		0.073		
**Non-cardio-cerebrovascular mortality in all people**							
Continuous	1.95(1.88,2.03)	<0.001	1.59(1.5,1.68)	<0.001	1.47(1.36,1.58)	<0.001	2188
Q1	Reference	-	Reference	-	Reference	-	165
Q2	1.66(1.37,2.02)	<0.001	1(0.83,1.22)	0.975	1.04(0.84,1.28)	0.725	271
Q3	3.7(3.12,4.41)	<0.001	1.37(1.15,1.65)	<0.001	1.42(1.17,1.73)	<0.001	558
Q4	11.14(9.49,13.15)	<0.001	2.04(1.71,2.45)	<0.001	2.04(1.69,2.49)	<0.001	1194
P for trend	<0.001		<0.001		<0.001		
**All-cause mortality in people with stroke**							
Continuous	1.8(1.57,2.02)	<0.001	1.69(1.42,1.96)	<0.001	1.44(1.15,1.77)	0.002	258
Q1	Reference	-	Reference	-	Reference	-	45
Q2	0.76(0.34,1.88)	0.529	0.69(0.31,1.73)	0.412	0.65(0.28,1.69)	0.364	58
Q3	1.5(0.74,3.53)	0.281	1.17(0.57,2.76)	0.693	1.14(0.53,2.78)	0.754	74
Q4	2.83(1.46,6.45)	0.001	1.42(0.72,3.29)	0.335	1.41(0.7,3.32)	0.359	81
P for trend	<0.001		0.007		0.008		

Model 1: no covariates were adjusted. Model 2: age, gender, and race were adjusted. Model 3: age, gender, race, educational level, family income-to-poverty ratio, BMI, hypertension, diabetes, chronic kidney disease, and smoking status.

Table S6. Mortality Risk Stratification by Youden's Index-Derived Cystatin C Thresholds.

	Model 1		Model 2		Model 3		Number of deaths
	HR [95% CI]	*P*-value	HR [95% CI]	*P*-value	HR [95% CI]	*P*-value	
**All-cause mortality in all people**							
Cystatin C ≤0.909	Reference	-	Reference	-	Reference	-	1528
Cystatin C > 0.909	6.51(5.78,7.34)	<0.001	1.94(1.76,2.14)	<0.001	1.77(1.61,1.94)	<0.001	1679
**Cardiovascular mortality in all people**							
Cystatin C ≤0.868	Reference	-	Reference	-	Reference	-	287
Cystatin C > 0.868	7.67(6.31,9.33)	<0.001	2.1(1.76,2.5)	<0.001	1.62(1.35,1.95)	<0.001	563
**Cerebrovascular mortality in all people**							
Cystatin C ≤0.817	Reference	-	Reference	-	Reference	-	47
Cystatin C > 0.817	6.42(4.08,10.1)	<0.001	1.63(1.06,2.51)	0.027	1.44(0.9,2.31)	0.13	122
**Non-cardio-cerebrovascular mortality in all people**							
Cystatin C ≤0.949	Reference	-	Reference	-	Reference	-	1233
Cystatin C > 0.949	6.68(5.85,7.63)	<0.001	1.98(1.78,2.21)	<0.001	1.84(1.63,2.07)	<0.001	955
**All-cause mortality in people with stroke**							
Cystatin C ≤0.996	Reference	-	Reference	-	Reference	-	119
Cystatin C > 0.996	2.45(1.75,3.42)	<0.001	1.38(1.03,1.85)	0.031	1.1(0.76,1.59)	0.614	139

Model 1: no covariates were adjusted. Model 2: age, gender, and race were adjusted. Model 3: age, gender, race, educational level, family income-to-poverty ratio, BMI, hypertension, diabetes, chronic kidney disease, and smoking status. Cut-off selection used maximizing youden's index.

Table S7. The mortalities in basic characteristics.

Characteristic	All-cause mortality in all people N = 3,207	All-cause mortality in people with stroke N = 258	Cardiovascular mortality in all people N = 850	Cerebrovascular mortality in all people N = 169	Non-cardio-cerebrovascular mortality in all people N = 2,188
Gender, %					
Female	45.09	42.64	43.18	48.52	45.57
Male	54.91	57.36	56.82	51.48	54.43
Age (Years), Mean±SD	68.61±13.68	71.92±10.92	70.76±12.60	71.15±12.56	67.58±14.04
Race/Ethnicity, %					
Mexican American	17.31	17.05	14.71	18.93	18.19
Non-Hispanic Black	17.06	16.67	17.41	14.79	17.09
Non-Hispanic White	60.34	60.85	63.53	60.36	59.1
Other Hispanic	3.09	2.33	2.47	4.14	3.24
Other Race or Multi-Racial	2.21	3.1	1.88	1.78	2.38
Education level, %					
High School	23.79	20.54	22.94	20.12	24.41
Less Than High School	42.59	49.61	43.88	47.93	41.68
More Than High School	33.61	29.84	33.18	31.95	33.91
Family income-to-poverty ratio (PIR), Mean±SD	2.34±1.47	2.09±1.34	2.26±1.44	2.31±1.39	2.38±1.49
BMI (kg/m^2), Mean±SD	28.17±5.93	28.80±5.64	28.55±6.10	27.90±4.99	28.05±5.92
Smoking, %	59.28	56.59	58.35	52.66	60.15
Diabetes, %					
Borderline	2.31	4.26	2.94	2.37	2.06
No	77.89	68.22	75.29	79.29	78.79
Yes	19.8	27.52	21.76	18.34	19.15
Hypertension, %	53.48	73.64	60.24	57.99	50.5
Coronary heart disease, %	11.19	20.54	16.24	8.28	9.46
Congestive heart failure, %	8.39	18.6	13.53	4.14	6.72
Emphysema, %	5.27	7.75	5.18	4.14	5.39
Chronic kidney dis-ease, %	5.86	12.79	7.53	4.73	5.3

Table S8. Model comparison using AIC and BIC criteria across different cystatin C specifications.

	Model 1			Model 2			Model 3		
	AIC	BIC	AIC/BIC	AIC	BIC	AIC/BIC	AIC	BIC	AIC/BIC
**Stroke mortality**									
Continuous	2457.69	2471.25	0.9945	2251.78	2311.12	0.9743	1991.23	2116.86	0.9407
Categorical	2295.96	2326.51	0.9869	2210.35	2283.15	0.9681	1968.68	2107.12	0.9343
**All-cause mortality in all people**									
Continuous	57637.48	57643.56	0.9999	52376.70	52419.21	0.9992	46526.68	46622.27	0.9979
Categorical	55739.01	55757.23	0.9997	52409.94	52464.59	0.9990	46502.03	46609.58	0.9977
**Cardiovascular mortality in all people**									
Continuous	15212.47	15217.22	0.9997	13501.45	13534.67	0.9975	12081.71	12156.22	0.9939
Categorical	14608.14	14622.38	0.9990	13540.33	13583.04	0.9969	12092.90	12176.72	0.9931
**Cerebrovascular mortality in all people**									
Continuous	3037.54	3040.67	0.9990	2699.44	2721.35	0.9919	2377.70	2426.09	0.9801
Categorical	2930.93	2940.32	0.9968	2704.4	2732.57	0.9897	2380.38	2434.81	0.9776
**Non-cardio-cerebrovascular mortality in all people**									
Continuous	39392.19	39397.88	0.9999	36156.38	36196.21	0.9989	32056.23	32145.66	0.9972
Categorical	38194.2	38211.27	0.9996	36150.93	36202.15	0.9986	32020.25	32120.85	0.9969
**All-cause mortality in people with stroke**									
Continuous	2669.95	2673.50	0.9987	2575.79	2600.66	0.9904	2256.35	2311.50	0.9761
Categorical	2650.65	2661.30	0.9960	2584.46	2616.44	0.9878	2257.92	2319.96	0.9733

Model 1: no covariates were adjusted. Model 2: age, gender, and race were adjusted. Model 3: age, gender, race, educational level, family income-to-poverty ratio, BMI, hypertension, diabetes, chronic kidney disease, and smoking status. AIC, Akaike Information Criterion; BIC, Bayesian Information Criterion.

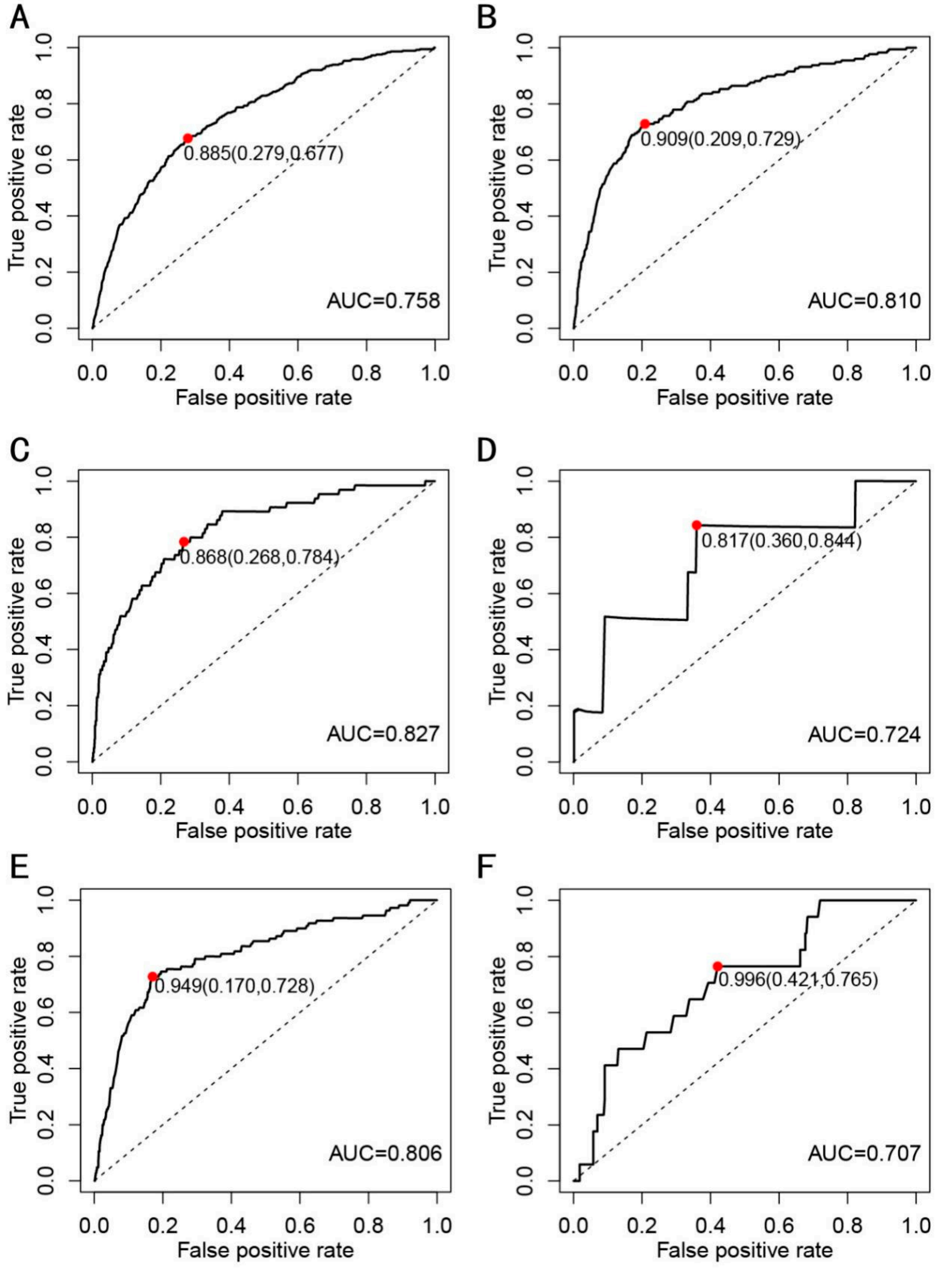

Figure S1. The ROC curve utilized in assessing the predictive capability of Cystatin C for stroke morbidity and mortality. (**A**) ROC curve for stroke morbidity. (**B**) ROC curve for all-cause mortality in all people. (**C**) ROC curve for cardiovascular mortality in all people. (**D**) ROC curve for cerebrovascular mortality in all people. (**E**) ROC curve for non-cardio-cerebrovascular mortality in all people. (**F**) ROC curve for all-cause mortality in people with stroke.
